# Studies of Fracture Toughness in Concretes Containing Fly Ash and Silica Fume in the First 28 Days of Curing

**DOI:** 10.3390/ma14020319

**Published:** 2021-01-09

**Authors:** Grzegorz Ludwik Golewski, Damian Marek Gil

**Affiliations:** 1 Department of Structural Engineering, Faculty of Civil Engineering and Architecture, Lublin University of Technology, Nadbystrzycka 40 Str., 20-618 Lublin, Poland; 2 Civil Engineering Laboratory, Faculty of Civil Engineering and Architecture, Lublin University of Technology, Nadbystrzycka 40 Str., 20-618 Lublin, Poland; d.gil@pollub.pl

**Keywords:** concrete, fly ash (FA), silica fume (SF), fracture toughness, curing time, compressive strength, splitting tensile strength, critical stress intensity factor

## Abstract

This paper presents the results of the fracture toughness of concretes containing two mineral additives. During the tests, the method of loading the specimens according to Mode I fracture was used. The research included an evaluation of mechanical parameters of concrete containing noncondensed silica fume (SF) in an amount of 10% and siliceous fly ash (FA) in the following amounts: 0%, 10% and 20%. The experiments were carried out on mature specimens, i.e., after 28 days of curing and specimens at an early age, i.e., after 3 and 7 days of curing. In the course of experiments, the effect of adding SF to the value of the critical stress intensity factor—$K_{\mathrm{Ic}}^{S}$ in FA concretes in different periods of curing were evaluated. In addition, the basic strength parameters of concrete composites, i.e., compressive strength—*f*_cm_ and splitting tensile strength—*f*_ctm,_ were measured. A novelty in the presented research is the evaluation of the fracture toughness of concretes with two mineral additives, assessed at an early age. During the tests, the structures of all composites and the nature of macroscopic crack propagation were also assessed. A modern and useful digital image correlation (DIC) technique was used to assess macroscopic cracks. Based on the conducted research, it was found the application of SF to FA concretes contributes to a significant increase in the fracture toughness of these materials at an early age. Moreover, on the basis of the obtained test results, it was found that the values of the critical stress intensity factor of analyzed concretes were convergent qualitatively with their strength parameters. It also has been demonstrated that in the first 28 days of concrete curing, the preferred solution is to replace cement with SF in the amount of 10% or to use a cement binder substitution with a combination of additives in proportions 10% SF + 10% FA. On the other hand, the composition of mineral additives in proportions 10% SF + 20% FA has a negative effect on the fracture mechanics parameters of concretes at an early age. Based on the analysis of the results of microstructural tests and the evaluation of the propagation of macroscopic cracks, it was established that along with the substitution of the cement binder with the combination of mineral additives, the composition of the cement matrix in these composites changes, which implies a different, i.e., quasi-plastic, behavior in the process of damage and destruction of the material.

## 1. Introduction

In accordance with the principles of sustainable construction, attempts should be made to minimize the use of ordinary Portland cement (OPC) for the production of concrete by replacing it with other materials. Such actions, which are undoubtedly pro-ecological, directly affect:

- reduction of greenhouse gas emissions (GHG) to the atmosphere, such as CO_2_, NO, NO_2_ [1,2,3];
- reduction of heat and electricity consumption [4,5,6];
- reduction of the extraction of natural resources [7,8,9];
- possibility to utilize industrial wastes, such as fly ash (FA) or silica fume (SF) [10,11,12].

Currently, many research centers conduct tests on the separate use of SF [13,14,15,16] and FA [17,18,19,20] for concrete and studies assessing the properties of composites for which the addition of both materials was used [21,22,23,24]. Innovative hybrid techniques are increasingly used based on the synergy of interconnected components. Examples of modern materials are binary, ternary, quaternary or even quinary binders with mineral additives [25,26,27,28,29,30,31,32,33,34,35,36,37,38,39]. Such materials also include composites that are the subject of this article, i.e., FA cements with the addition of SF, e.g., [40].

It should be noted that several basic properties of OPC–FA–SF concrete, such as consistency, setting time, workability, sorptivity, compressive strength, durability and resistance to migration corrosive substances, were provided so far in papers [41,42,43,44,45,46,47,48]. During these investigations, both ordinary and high strength concrete were also analyzed, e.g., [49]. However, the potential of FA and SF as substitutes of binder in concrete composites were presented in detail in [50]. The article presents the results of research describing the beneficial effect of combined additives on selected mechanical parameters (compressive strength and splitting tensile strength) and physical parameters (water absorption, depth on water penetration under pressure, frost resistance). The work by [51] presents the results of fracture toughness of concretes made with hybrid binders, i.e., with the combined additive of FA and SF. Unfortunately, the presented results of the experiments covered only the tests of mature concretes.

Furthermore, in papers [21,48,49], the results of fracture toughness tests of concretes containing FA and SF, conducted under the Mode I fracture, were presented only for mature composites. The experiments testing the ordinary [21,48] and high-performance concretes [49] were presented in those papers. In these publications, the following parameters of the fracture mechanics were analyzed: critical stress intensity factor, fracture energy, effective crack length and critical crack tip opening displacement. The relational curves between the vertical load and the mid-span deflection (*P_V_*-δ), crack mouth opening displacement (*P_V_*-*CMOD*) and crack tip opening displacement (*P_V_*-*CTOD*) were also analyzed in papers [21,49]. Based on the test results presented in these publications, for mature concretes, it was found that, among others, low volumes of FA, i.e., 10% improve the fracture mechanics parameters of concrete and a small amount of SF, i.e., 5%, has a positive effect on fracture toughness.

However, from previous reports, it is known that the addition of FA to concrete has a positive effect on the parameters of fracture mechanics of 28-day and older concretes and a negative effect on the properties of composites at a young age [52,53,54,55,56,57,58,59,60,61,62]. This disadvantageous property of the material could be improved by substituting the cement binder FA in combination with SF.

Therefore, in order to supplement the previous information included in papers [21,49,50,51], this article analyses the fracture toughness of concretes containing three types of binder, i.e., OPC, FA and SF in the period between 3rd and 28th day of curing. In the scope of additional tests, it was also analyzed how the basic strength parameters of the composites changed in the analyzed time interval. A novelty in the presented research is the evaluation of the fracture toughness of concretes with two mineral additives, assessed at an early age. Moreover, it should be noted that the fracture toughness tests for concretes at an early age with hybrid binders, i.e., containing both FA and SF additives, have not been the subject of an in-depth analysis so far.

## 2. Purpose and Scope of the Experimental Research

Microcracks that may appear in the structure of concrete at an early age negatively affect the progress of the final strength of the composite and reduce its fracture toughness. Therefore, it is important to track how this important property of the material changes, i.e., fracture toughness from the beginning of the formation of the composite structure until it is cured.

Moreover, they are also very important from a technical point of view. The early strength of concrete is extremely important in today’s construction industry. Due to the pace of construction and the number of investments in the area of road, service, residential and industrial infrastructure, time plays an extremely important role.

It should be noted that the knowledge of parameters of fracture mechanics for young concrete is particularly important in the following concrete construction areas:

- Monolithic massive constructions, where the young concrete is subjected to load increases during the subsequent stages of construction of the structure as a result of which numerous technical decisions must be made depending on the instantaneous strength of the concrete or its other properties, such as time of stripping the formwork, moving the sliding formwork, allowing young concrete to be subjected to technological and assembly loads, etc.;
- Prefabricated reinforced concrete constructions, where it is important to know the interoperational strength, e.g., formwork stripping, transport, storage, shipping, assembly;
- Prestressed reinforced concrete constructions, in which it is important to know the possible short-term losses of prestressing forces or not fully cured concrete.

Siliceous FA used in the amount of approx. 20% of the cement mass significantly improves the mechanical parameters of concrete, e.g., their fracture toughness after 28 days of curing. However, the disadvantage of this solution is the reduction of the early strength of concrete. The article attempts to increase the early strength of FA concretes using a second pozzolanic additive. The experimental tests were carried out in order to confirm the positive effect of the applied modifications on the early parameters of compressive and tensile strength as well as fracture toughness of concrete. The presented research results can be practically used in many areas of construction, where the pace of construction works plays a decisive role. Moreover, the obtained test results may be helpful in selecting the composition of concrete mixtures containing FA and SF to be used in concrete and reinforced concrete structures subjected to loads at an early age.

Therefore, in order to assess strength parameters and parameters of fracture mechanics of young concrete, the tests were conducted after 3, 7 and 28 days following the preparation of batches of concrete.

The parameters of concretes modified with FA in the amount of up to 30% have already been fully tested, e.g., [52,55,56]. The beneficial effects of modifying concrete composites by SF in an amount up to 10%, e.g., [15,21,50], are also well known. However, in the presented experiments, it is planned to examine the interaction of pozzolanic additives and to assess their synergies in relation to the improvement of the fracture toughness of concretes containing ternary binders, e.g., [27,38,40]. For this purpose, we proposed proprietary concrete mixes, which had not been used before.

During the experiments, it is planned to investigate changes in concrete compressive strength (*f*_cm_), splitting tensile strength (*f*_ctm_) and fracture toughness of young and mature concretes containing two pozzolanic additives, i.e., FA and SF. The analyses took into account the basic parameter of concrete fracture toughness determined under Mode I fracture. The parameter of fracture toughness assessed in the tests was a critical stress intensity factor ($K_{\mathrm{Ic}}^{S}$.)

However, there are many measurement techniques for the evaluation of fracture properties of cementitious composites, e.g., [63,64,65,66,67,68,69,70,71,72,73,74]. On their basis, it was found that microcracks develop in concrete structures during the loading process, which leads to a gradual loosening of the material. Thus, fracture toughness of brittle materials should be examined under various load states, e.g., [75,76,77,78,79,80,81,82].

## 3. Experimental Procedure

### 3.1. Materials

The basic binder used in the experiments was OPC, CEM I 32.5 R from Chełm cement plant, with: compressive strength equal to 23.3 MPa after two days and 50 MPa after 28 days of curing. As a substitute of OPC binder FA, from a local power plant, as a result of energetic combustion of hard coal in the Puławy thermoelectric power station and noncondensed SF from Łaziska Ironworks were used.

The chemical composition of the binders used is given in Table 1, whereas their essential physical parameters are given in Table 2. Additionally, Table 3 presents a division into the fraction of SF and FA used and the average particle diameter of both materials.

The chemical composition of both additives was determined by the XRF method. A Epsilon 3X spectrometer was applied (Malvern Panalytical, Malvern, UK). On the other hand, analyzed physical parameters were assessed as follows:

- Specific density by pycnometric method;
- Specific surface area according to the Blaine method;
- Particle size distribution by laser granulometry using measuring device Masterizer 3000 and measuring range 0.01–3500 μm were used (Malvern Panalytical, Malvern, UK);
- Loss of ignition by burning the individual materials for one hour at 1000 °C;
- Color—visually.

**Figure 1:**
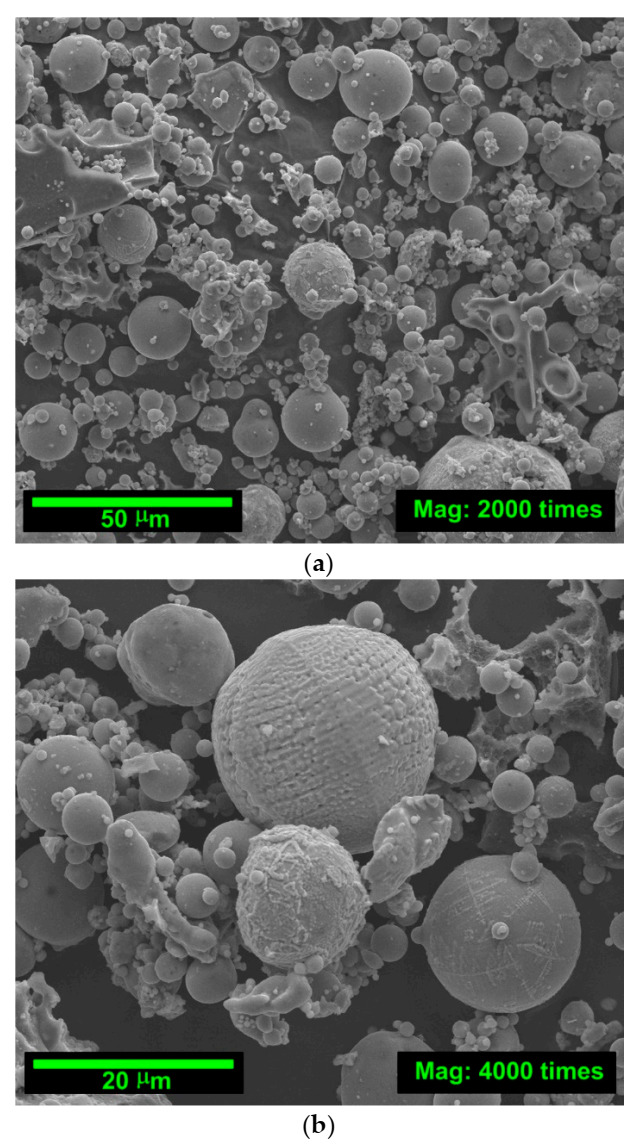

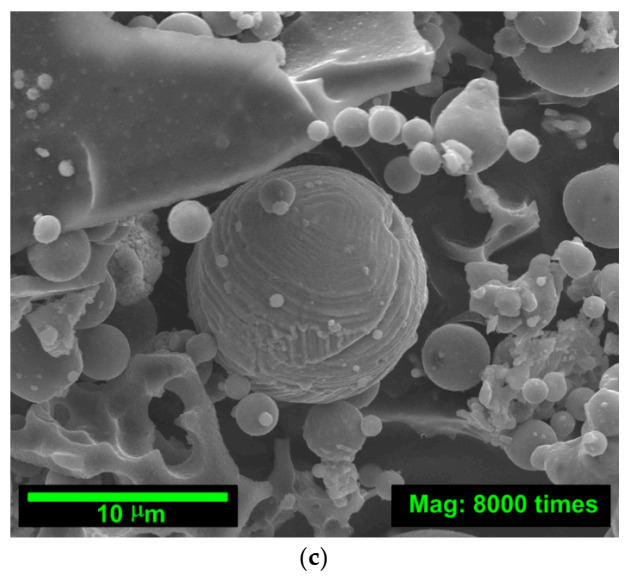
SEM images of siliceous FA: (**a**) magnification: 2000×; (**b**) magnification: 4000×; (**c**) magnification: 8000×.

**Figure 2:**
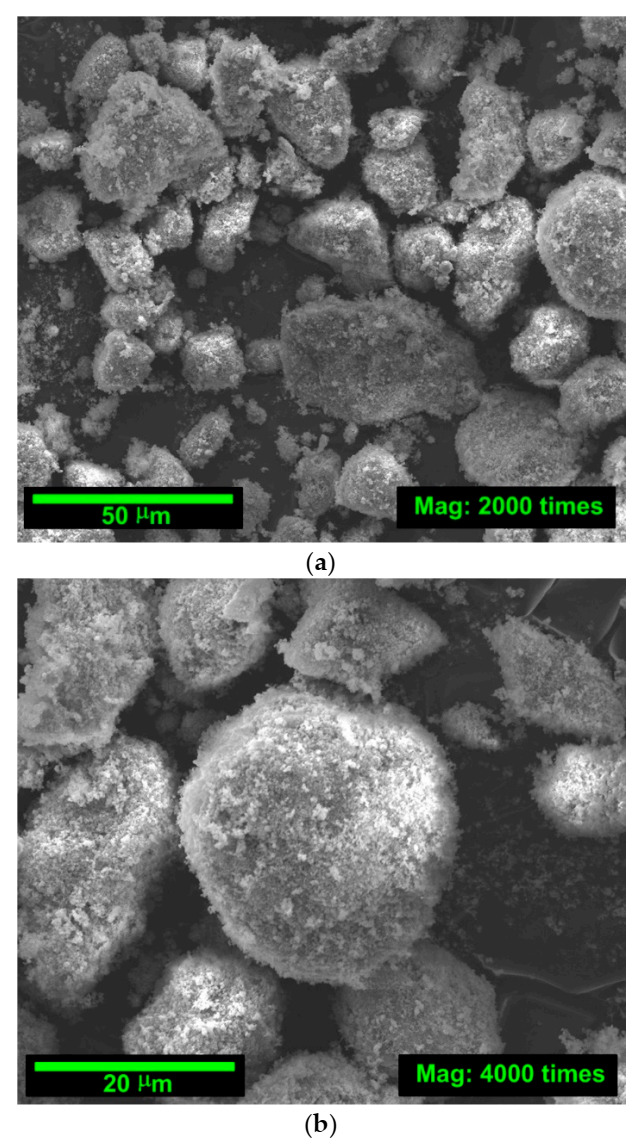

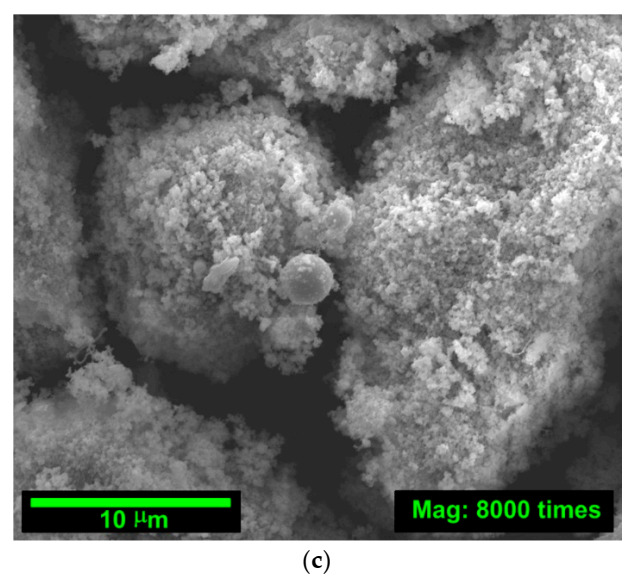
SEM images of noncondensed SF: (**a**) magnification: 2000×; (**b**) magnification: 4000×; (**c**) magnification: 8000×.

In addition, Figure 1 and Figure 2 shows the SEM pictures of the mineral additives used. The morphologies and the particle sizes of the cementitious materials can be observed in figures. In order to diagnose whether the particles of individual materials are homogenous and have similar grain morphology, each of the mineral additives is shown at three magnifications, i.e., 2000, 4000 and 8000 times (Figure 1 and Figure 2).

Based on the pictures shown in Figure 1 and Figure 2, it can be concluded that SF of the finest size, followed by FA (Figure 2). However, the size of FA particles was also fine grading with well-developed surfaces (Figure 1). The small particle size of the SF and FA have a beneficial effect on the parameters of strength and fracture mechanics as well as physical parameters of concretes with these additives, e.g., [83,84,85].

On the basis of the observations of SEM FA pictures shown in Figure 1, it can be additionally stated that the vast majority of grains have regular and spherical shapes with glazed surfaces. High homogeneity and uniform distribution of particles were observed, which is confirmed by the results of the particle size analysis given in Table 3.

The observation of SF grains, shown at 3 different magnifications in Figure 2, reveals that the grains of this pozzolanic additive are regular in shape and are made of very fine silica particles. Their surface is matte and rough. In the case of SF image analysis, similarly, as with the SEM FA, a distinct homogeneity was observed in the structure and morphology of the grains of this material.

Moreover, 0–2 mm pit sand as fine aggregate, 2–8 mm gravel as coarse aggregate, pipeline water and plasticizer were used to prepare concrete mixtures. The tests assessed the combined effect of the addition of both wastes, i.e., FA and SF. SF had a constant amount of the additive, i.e., 10%, the amount of FA was varying. In each series of concrete, OPC was substituted with additives by weight.

Four concrete mixtures were used for testing the basic strength of materials characteristics and the fracture toughness, which are as follows:

- Mixture without additives (FA00+SF00);
- Mixture without FA additive and with a 10% SF additive (FA00+SF10);
- Mixture with a 10% FA additive and with a 10% SF additive (FA10+SF10);
- Mixture with a 20% FA additive and with a 10% SF additive (FA20+SF10).

The detailed composition of the above concrete mixtures was given in [50].

For each type of concrete mixture and for each curing time, the following were made:

- 6 cube specimens (150 mm) for compressive strength tests—*f*_cm_, according to PN-EN12390-3 [86];
- 6 cube specimens (150 mm) for splitting tensile strength tests—*f*_ctm_, according to PN-EN12390-6 [87];
- 6 beams (80 × 150 × 700 mm with one initial crack) for fracture toughness tests at Mode I fracture—$K_{\mathrm{Ic}}^{S}$, according to RILEM draft recommendation [88].

All tests were carried out after 3, 7, and 28 days of curing.

### 3.2. Methods

Both compression and tensile strength were tested using a compression machine (Walter + Bai ag) with a maximum load of 3000 kN. The specimens were loaded statically.

**Figure 3:**
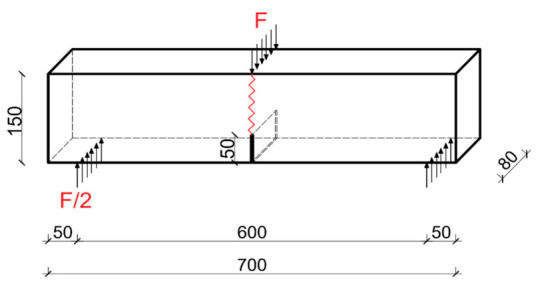
A scheme of specimen used in the basic studies; *F*—force, dimensions in (mm).

Fracture toughness tests, under Mode I fracture, were conducted in accordance with the draft guidelines of RILEM recommendations [88]. The results of the critical stress intensity factor were analyzed during the tests. The beams with dimensions of 700 × 150 × 80 mm, which one initial crack in the center was used to assess the fracture toughness of concrete under Mode I fracture. During the experiments, the force was applied in the middle of the span of the beam, e.g., [89]. A schematic illustration of the specimen with dimensions and loading conditions is shown in Figure 3.

**Figure 4:**
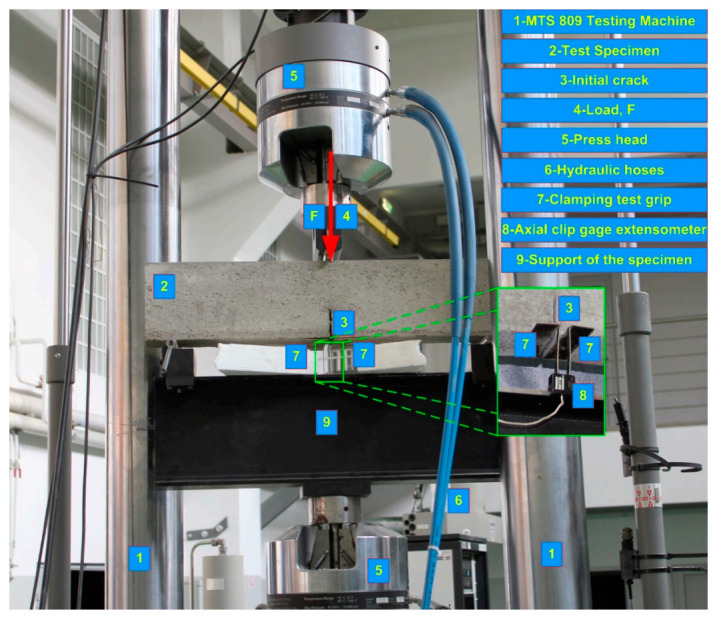
View of experimental stand for testing according to Mode I fracture.

The research results were collected thanks to the recording of the results from the press (Materials Test System, MTS; type 809; MTS Systems Corp.; Eden Prairie, MN, USA) and clip gauge axial extensometer, which was placed on the clamping test grips. The test setup apparatus with all details is presented in Figure 4.

**Figure 5:**
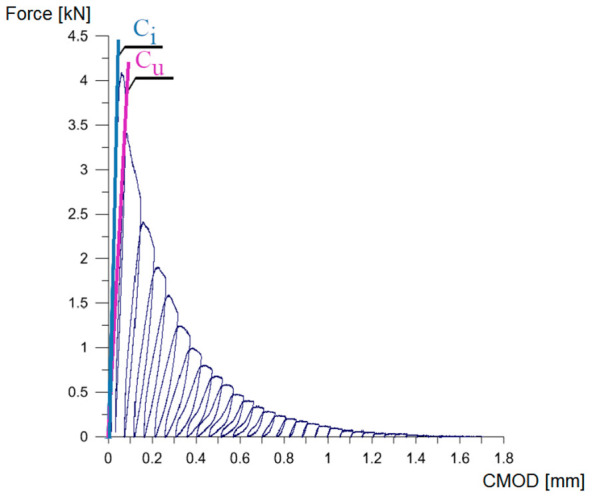
An example graph of crack mouth opening displacement (CMOD)-load relationship obtained in the tests under Mode I fracture: *C*_i_, *C*_u_—description in the text.

For each of the tested cuboidal specimens (Figure 3), the load (*F*)–crack mouth opening displacement (*CMOD*) curve was recorded. The exemplary curve of *F*–*CMOD* is shown in Figure 5. Thanks to these graphs and the equations given in [90], the fracture mechanics parameters of individual concretes were determined.

It should be noted that the most important data obtained for each of the graphs were (Figure 5):

- Maximum load obtained in the tests;
- Tangent in the first phase on the *F*–*CMOD* relationship, highlighted in blue (*C*_i_) in Figure 5;
- Tangent in the second phase on the *F*–*CMOD* relationship, highlighted in purple (*C*_u_) in Figure 5.

Additionally, the digital image correlation (DIC) technique was used to assess the shape and trajectory of the crack paths in particular concretes. This modern and increasingly often used measurement method allows for accurate and precise tracking of the cracking processes on the surface of concrete elements during the progressive crack propagation process. Thanks to it, it is possible to distinguish subtle differences in the shape of macrocracks depending on changes in the composition of the tested concretes. Such experiments provide valuable information and are a valuable supplement to the test results of concrete mechanical parameters.

## 4. Results and Discussions

### 4.1. Mechanical Properties

**Figure 6:**
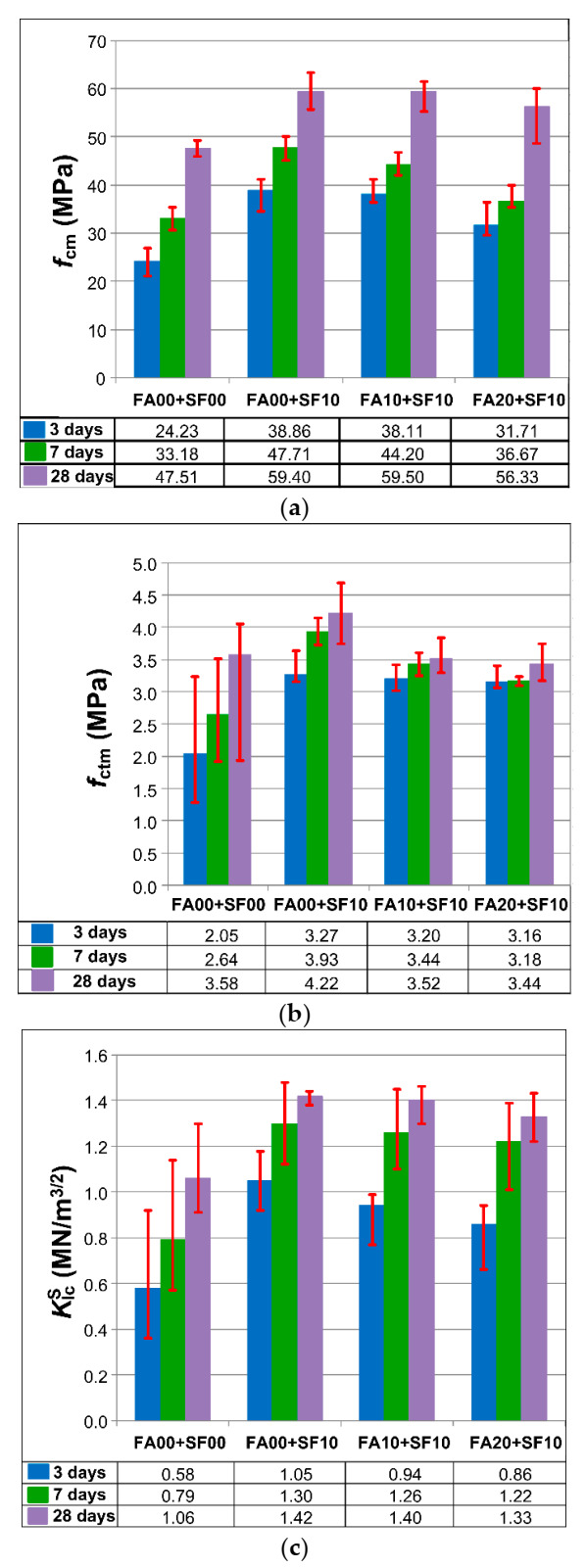
The results of the analyzed parameters of concretes: (**a**) compressive strength, (**b**) splitting tensile strength, (**c**) fracture toughness at Mode I.

The average values of the analyzed mechanical parameters for particular periods of curing (with error bars) are given in Figure 6. In addition, Table 4 presents the percentage increases of 3 analyzed mechanical parameters in 3 periods of curing in relation to the values that were obtained for the concrete without additives.

Based on the results presented in Figure 6, it was observed that the highest indices of all mechanical parameters in all periods of curing had concrete containing only SF. The most significant effect of using this additive was noticeable in the youngest concrete, i.e., 3-day concrete. Therefore, an increase of approx. 60% in both strengths and 80% in fracture toughness were observed in comparison to the reference concrete; FA00+SF10 (Table 4). The other two modified matrix composites also had higher mechanical parameters when compared to FA00+SF00 concrete. Similar trends, although with a less pronounced dominance of the FA00+SF10 mixture in the obtained results, were observed in the concrete after 7 and 28 days of curing. It should also be noted that, in all series of concrete with ternary binders, the results of the analyzed fracture mechanics parameter, i.e., critical stress intensity factor, clearly exceeded the values of $K_{\mathrm{Ic}}^{S}$ that were obtained for unmodified concrete, i.e., FA00+SF00. In the case of this parameter, the following increases were obtained:

- From 48% to 81% (after 3 days);
- From 54% to 65% (after 7 days);
- From 26% to 34% (after 28 days).

The positive changes in the mechanical properties of concretes containing fine and chemically active mineral additives (such as those used in our own research, i.e., FA and SF) are directly related to the structural changes occurring in the composites. It has been proven in numerous papers to date that these materials significantly modify the microstructure of concrete with a changed cement binder composition, which translates into an improvement in their mechanical parameters. This phenomenon applies to ordinary concretes, e.g., [90,91,92,93,94], and high-performance concretes, e.g., [95,96].

Due to the chemical composition (Table 1), physical parameters (Table 2), fine graining (Table 3) and the grain morphology of the micro-fillers used (Figure 1 and Figure 2), the addition of SF is particularly beneficial in the early curing periods of the material. In the later dates of testing, however, the benefits of the substitution of cement by FA are also noticeable. Taking into account this information, attempts were made to explain the results obtained in the strength tests based on the analysis of changes occurring in the microstructure of individual composites in the subsequent periods of their maturation. The results of these studies are presented in the next subsection.

### 4.2. Microstructure of Composites

**Figure 7:**
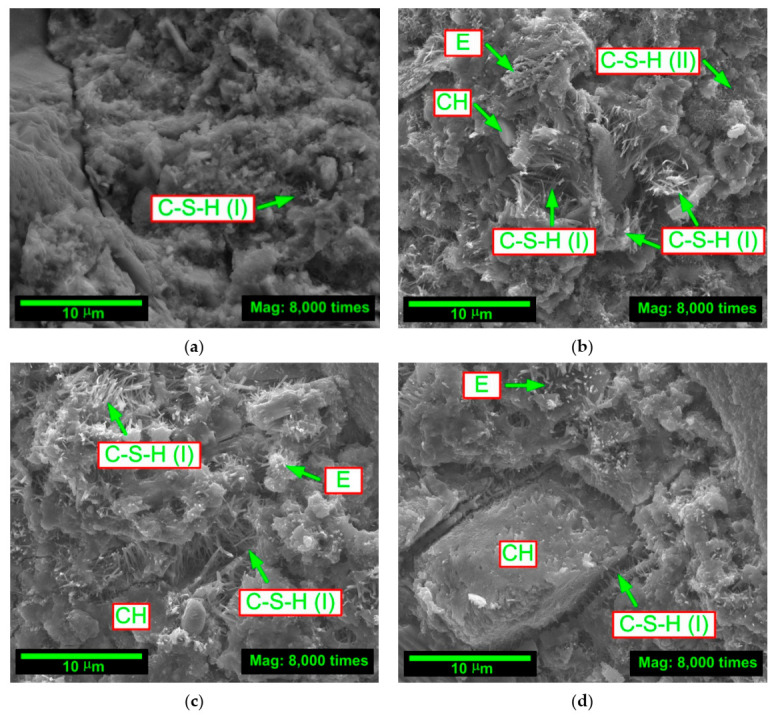
Microstructure of analyzed concretes after 3 days of curing: (**a**) FA-00+SF-00; (**b**) FA-00+SF-10; (**c**) FA-10+SF-10; (**d**) FA-20+SF-10.

**Figure 8:**
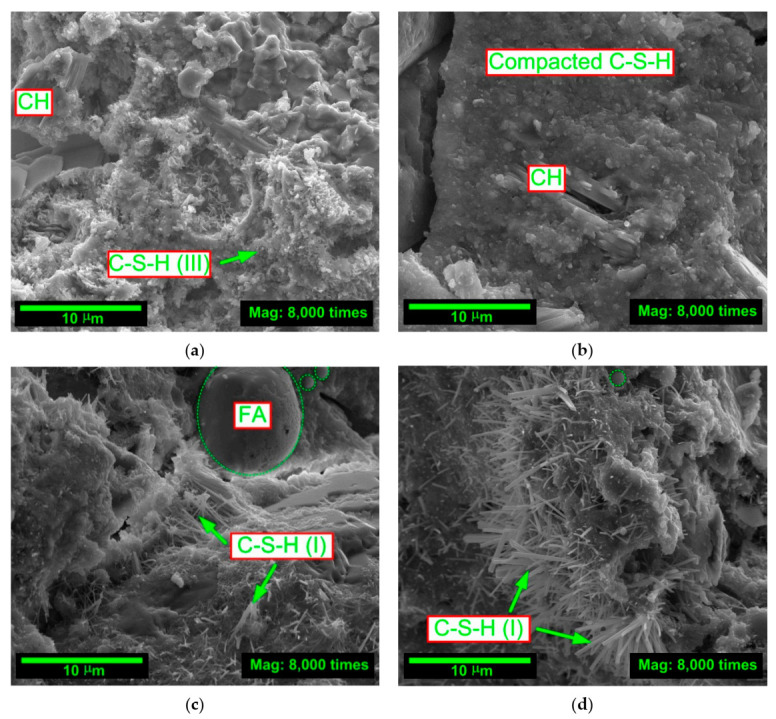
Microstructure of analyzed concretes after 28 days of curing: (**a**) FA-00+SF-00; (**b**) FA-00+SF-10; (**c**) FA-10+SF-10; (**d**) FA-20+SF-10.

Due to the undeniable relationship between the microstructure of concrete composites modified with mineral additives and the mechanical parameters of these materials, i.e., the processes of formation and propagation of microcracks, e.g., [97,98], an attempt was made to explain the results from the macroscopic fracture toughness tests—for particular series of concrete—based of the assessment of their structures. The pictures analyzing the type and intensity of phases occurring in concretes were taken for this purpose. The microstructural analysis was conducted with the use of the application of the scanning electron microscope (SEM) to assess the morphology of the fractured concrete surfaces. The microstructural testing was carried out using a QUANTA FEG 250. The first and last of the assessed time periods were taken into account. Microstructures of the analyzed concretes after 3 and 28 days of curing are shown in Figure 7 and Figure 8, respectively. In order to visualize the differences between particular materials as much as possible, the image magnification, i.e., 8000 times, and the reference scale, i.e., 10 μm (similarly as in SEM images from Figure 1c and Figure 2c showing both used additives), were the same in all cases. Additionally, Figure 7 and Figure 8 show the locations of the main phases in the concrete that affect its mechanical parameters.

A high quantity of the hydration products in the form of ettringite (E) and calcium silicate hydrate of form I (C–S–H (I)) in FA-00+SF-10 and FA-10+SF-10 concretes can be seen in Figure 6c and Figure 7b. In concrete containing only SF, even the C-S-H phase of form II (C-S-H(II)) was observed, i.e., it looks like a honeycomb. Moreover, in these concretes, calcium hydroxide (CH) could also be observed, but to a lesser extent than it was the case for the other two main phases. However, large portlandite crystals, weakening the structure of the material, were diagnosed in 3-day old FA-20+ SF-10 concrete.

After four weeks, the FA-00+SF-10 and FA-10+SF-10 concretes already had a compact structure with barely visible CH crystals. This is due to the pozzolanic reaction that occurred between SF and CH, which led to a reduction in CH. Furthermore, in concretes containing FA—after 28 days of curing—the FA particle of approximately 8 μm and 2 μm were observed (circled with green dashed lines) (Figure 8c,d). Moreover, in the reference concrete, in both of the analyzed time periods, the cement matrix phases were weakly developed (Figure 7a and Figure 8a).

The rapid development of the C-S-H phase after only 3 days of curing in FA-00+SF-10 concrete (Figure 7b) and FA-10+SF-10 concrete (Figure 7c), and its clear transformation in these composites into a rigid matrix in the last of the analyzed time periods (Figure 8b,c) caused that the concretes of these two series had clearly higher values of fracture toughness compared to other composites (Figure 6c). The concrete containing a greater amount of FA, after 3 and 28 days, still had a disordered structure, and even after 4 weeks, areas of the C-S-H (I) phase at an early stage of the development were visible in it (Figure 8d). As a result, this concrete was also characterized by worse technical parameters. However, the composite without additives (Figure 7a and Figure 8a) had by far the least developed phases in the matrix structure. Due to the lack of active modifiers (mainly SF), the concrete of this series, in all analyzed time periods, was characterized by the lowest values of the parameter $K_{\mathrm{Ic}}^{S}$.

The above assessment of the microstructure of concretes with a modified composition leads to the conclusion that the combination of chemically active and very fine-grained pozzolanic additives in the form of FA and SF clearly affects changes in the structure of composites at a young age and in the mature period. This is mainly reflected in the nucleation of additional regions of the C-S-H phase, which changes its structure from disordered to compact as the maturation process progresses (Figure 7 and Figure 8). It is particularly noticeable in FA-00+SF-10 and FA-10+SF-10 concretes. The content of microfillers also reduces the amount and, above all, the size of the pores present in the structure of the cement matrix. The development of additional phases (mainly C-S-H) through the use of FA and SF causes that they are located in the voids of the composite, gradually filling them, e.g., [99]. Due to the reduced hydration heat of concretes with mineral additives, the processes of formation of the first shrinkage cracks in the concrete structure are also reduced, e.g., [100,101,102,103]. As a consequence, the above effects lead to the homogenization of the structure of these materials, the increase in their stiffness and the improvement of mechanical parameters (see Section 4.2.). In addition, such processes may also decide about the improvement of the concrete’s fracture toughness and the change in the propagation of macroscopic cracks formed during its process of failure under the influence of external loads. These issues are discussed in detail in Section 4.3.

### 4.3. Toughness and Macrocrack Propagation

In addition to the microstructural analysis, an in-depth evaluation of the macroscopic cracks formed in the specimens after the tests were conducted. For this purpose, diagnostics with the naked eye and with the use of up-to-date and precise digital image correlation (DIC) technique were used.

**Figure 9:**
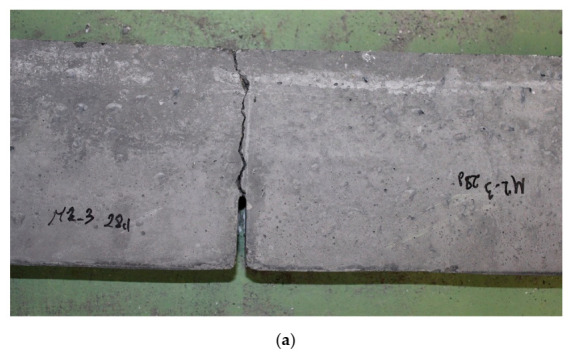

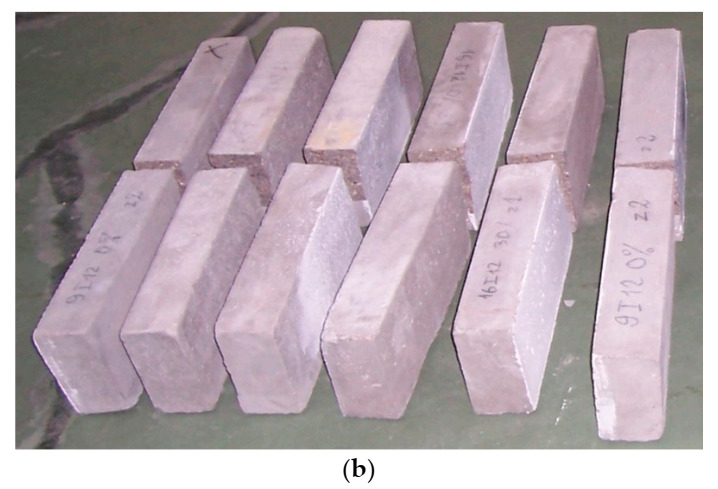
View of beams after conducted tests: (**a**) single beam with a characteristic type of failure, (**b**) batch of beams of series FA-10+SF-10.

Figure 9 shows a view of tested specimens after fracture toughness tests. Figure 9a shows a single beam after conducted the experimental test, while Figure 9b depicts a batch of fractured six specimens of series FA-10+SF-10.

The beams were usually damaged as a result of a single large crack (Figure 7a), similar to those observed in [86,104]. This phenomenon was more rapid in concrete without additives, while the modified beams were characterized by a quasi-plastic failure mode.

**Figure 10:**
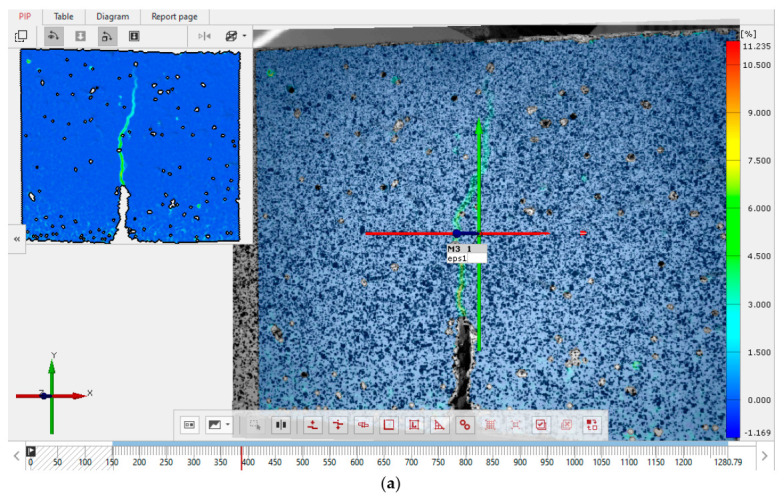

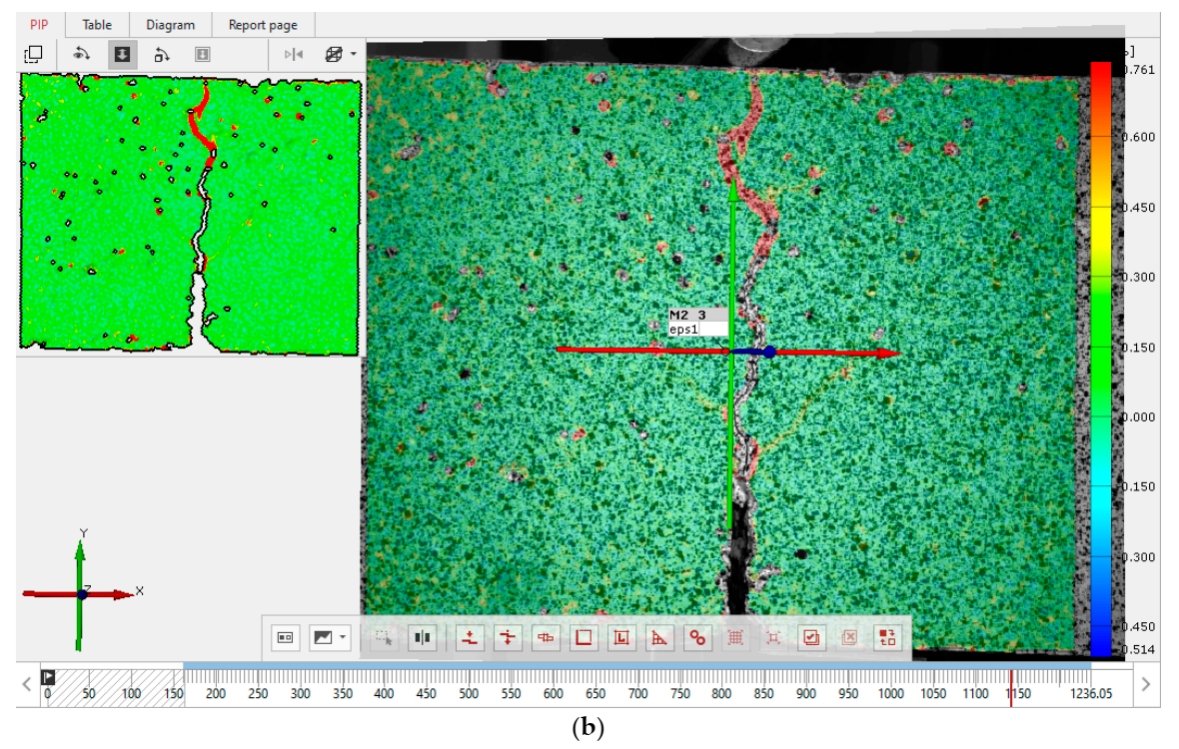
Examples of crack shapes observed in the tests using the digital image correlation (DIC) method: (**a**) quasi-straight crack for FA-00+SF-00 concrete; (**b**) curved crack for FA-20+SF-10 concrete.

This type of crack development is also confirmed by the results of experiments carried out with the use of the DIC technique [60,105,106,107]. The example of crack shapes observed in the tests using the DIC technique under Mode I fracture for concretes after 28 days of curing are shown in Figure 10. Figure 10a shows quasi-straight cracks, occurring mainly in the most brittle concrete, i.e., FA-00+SF-00, whereas Figure 10b shows curved cracks with branches, occurring in more plastic concretes (with additives), i.e., FA-20+SF-10.

The observed increase in the plasticity of concretes containing the combination of the mineral additives could probably be influenced by changes in their structure related to the slow development of additional phases in the cement matrix. The plasticity phenomenon was the most noticeable in FA-20+SF-10 concrete (Figure 10b). This composite was characterized by a structure with a high content of phases; however, only at the initial stage of their growth. Since higher FA content, i.e., 20% in the total composition of additives, caused a partial delay in the processes of homogenization of the cement matrix in the composite, FA-20+SF-10 concrete had the least compact structure and lower stiffness even after 28 days of curing (Figure 8d). This, in turn, reduced its brittleness and, during the process of macroscopic cracks propagation and destruction, revealed the quasi-plastic behavior (Figure 10b).

Additionally, more brittle forms of failure were observed in concretes with the shortest curing time, i.e., those tested three days after the specimens were formed. This is also confirmed by the results presented by other authors, e.g., [108,109,110]. Such a tendency was also observed in the tests of the fracture toughness of modified concretes, assessed according to Mode II and Mode III fracture, which authors had tested previously [17,19].

## 5. Conclusions

This paper investigates the behavior of ternary concretes after incorporating FA and SF. The main purpose of the new tests was to determine the effect of curing time of concretes modified with two additives on their fracture toughness assessed in accordance with Mode I fracture. During the experiments, the fracture toughness of young concretes (3 and 7 days old), and mature concretes (28 days old) were assessed.

The experimental results revealed that the addition of wastes, such as FA and SF in OPC, very positively affects the mechanical properties of concrete. Therefore, it is planned to continue the topic discussed in the article and to extend the tests on fracture toughness of concretes containing siliceous fly ash and noncondensed silica fume in terms of the analysis of fracture processes in these materials in complex stress states and the assessment of the microstructure of composites, after the process of their destruction.

Nonetheless, based on the obtained test results, the following conclusions can be drawn:

Both FA and SF grains are characterized by a homogeneous structure and a similar particle morphology. In the case of FA, the grains have regular and spherical shapes with glazed surfaces (Figure 1) with a uniform distribution of particles (Table 3). SF grains, on the other hand, have a regular shape and consist of very fine silica particles. Their surface is matte and rough (Figure 2). Their composition is dominated by very fine particles with grain size, mainly in the range of 0.01–20 μm (Table 3).

Concrete composites at an early age with siliceous fly ash and noncondensed silica fume in the cement composition obtain higher values of strength parameters (30% to over 60%) as well as fracture mechanics parameters (40% to over 80%) in comparison to the values obtained for plain concrete.

In all periods of curing, the maximum fracture toughness occurs in concrete containing only noncondensed silica fume. It is 1.05 MN/m^3/2^ after 3 days, 1.30 MN/m^3/2^ after 7 days and 1.42 MN/m^3/2^ after 28 days.

Substitution of the cement matrix with siliceous fly ash reduces the fracture toughness of concrete, mainly at an early age. This trend is more clearly noticeable in FA20+SF10 concrete.

All series of modified concretes have higher values of fracture toughness in all periods of curing compared to FA00+SF00 concrete.

A clear increase in values of the critical stress intensity factor is noticeable between the 3rd and 28th day of curing, particularly in the concrete from batches FA10+SF10 and FA20+SF10 (Figure 5). It means that, as the period of curing increases, the fracture toughness of ternary concretes—containing both siliceous fly ash and noncondensed silica fume—also increases.

FA00+SF-10 and FA-10+SF-10 concretes had the most developed phases in the structure of the cement matrix, in which C-S-H (I) and (E) dominated at a young age, while after 28 days, mainly the compact C-S-H phase dominated. Moreover, the structure of these concretes was more compact. In contrast, the reference concrete had less developed phases, while the composite with a greater amount of FA showed a distinct delay in growth and the transformation of the C-S-H (I) phase from a fibrous to a rigid form. It was also observed that the structures of these two materials were not fully ordered.

The process of crack development in the reference concrete was brittle while in modified concretes—quasi-plastic.

In the first 28 days of concrete curing, the preferred solution is to replace cement with SF in the amount of 10% or to use a cement binder substitution with a combination of additives in proportions 10% SF+10% FA.

The composition of mineral additives in proportions 10% SF + 20% FA has a negative effect on the fracture mechanics parameters of concretes at an early age.

## Figures and Tables

**Table 1:** Chemical composition of the ordinary Portland cement (OPC) and the additives.

Chemical	Component (wt %)		
	OPC	FA	SF
SiO_2_	15.00	55.27	91.90
Al_2_O_3_	2.78	26.72	0.71
Fe_2_O_3_	2.72	6.66	2.54
CaO	71.06	2.35	0.31
K_2_O	1.21	3.01	1.53
SO_3_	4.56	0.47	0.45
MgO	1.38	0.81	1.14
P_2_O_5_	-	1.92	0.63
TiO_2_	-	1.89	0.01
Ag_2_O	-	0.10	0.07
MnO	-	-	0.26
Cl	0.08	-	0.28

**Table 2:** Physical parameters of cementitious materials.

Property	Unit	Material		
		OPC	FA	SF
Specific density	(g/cm^3^)	3.23	1.90	2.21
Specific surface area	(cm^2^/g)	4294	2944	26,230
Loss of ignition	(%)	3.64	4.66	3.83
Color	-	Light gray	Dark gray	Black

**Table 3:** Division of the silica fume (SF) and siliceous fly ash (FA) into fractions.

Particle Size (μm)	Volume (%)	
	SF	FA
0.01−2	68.38	1.87
2−20	23.11	23.84
20−50	8.06	23.15
50−100	0.45	26.28
100−250	0.00	21.70
250−500	0.00	3.13
500−1000	0.00	0.03
1000−2000	0.00	0.00
Average particle diameter	10.666	102.035

**Table 4:** Relative changes in mechanical parameters of composites in relation to reference concrete.

Mix	Age (Days)	The Values of Analyzed Parameters Compared to FA00+SF00 (%)		
		*f*_cm_	*f*_ctm_	$K_{Ic}^{S}$
FA00+SF00	3	100	100	100
FA00+SF10		160.4	159.5	181.0
FA10+SF10		157.3	156.1	162.1
FA20+SF10		130.9	154.1	148.3
FA00+SF00	7	100	100	100
FA00+SF10		143.8	148.9	164.6
FA10+SF10		133.2	130.3	159.5
FA20+SF10		110.5	120.5	154.4
FA00+SF00	28	100	100	100
FA00+SF10		125.0	117.9	134.0
FA10+SF10		125.2	98.3	132.1
FA20+SF10		118.6	96.1	125.5

## Data Availability

No new data were created or analyzed in this study. Data sharing is not applicable to this article.
